# Extended survival observed in adoptive activated T lymphocyte immunotherapy for advanced lung cancer: results of a multicenter historical cohort study

**DOI:** 10.1007/s00262-012-1226-4

**Published:** 2012-03-16

**Authors:** Kazuro Iwai, Kenzo Soejima, Shoji Kudoh, Yoshimasa Umezato, Toru Kaneko, Kouji Yoshimori, Hitoshi Tokuda, Tetsuo Yamaguchi, Akira Mizoo, Yasuhiro Setoguchi, Takashi Kamigaki, Katsunada Fujimoto, Shigenori Goto

**Affiliations:** 1 Lung Cancer Immunotherapy Evaluation Group (LITEG), Tokyo, Japan; 2 Seta Clinic Group, Koushikai, 3-6-5, Iidabashi, Chiyoda-ku, Tokyo, 102-0072 Japan; 3 Department of Respiratory Medicine, School of Medicine, Keio University, Tokyo, Japan; 4 Fukujuji Hospital, Japan Anti-tuberculosis Association, Tokyo, Japan; 5 Department of Health Care Management Services, School of Medicine, Nihon University, Tokyo, Japan; 6 Social Insurance Chuo General Hospital, Tokyo, Japan; 7 JR Tokyo Central Hospital, Tokyo, Japan; 8 Tokyo Kosei Nenkin Hospital, Tokyo, Japan; 9 Department of Internal Medicine, Tokyo Medical University, Tokyo, Japan

**Keywords:** Advanced lung cancer, Adoptive immunotherapy, T lymphocyte, Overall survival, Additive effect, Performance status

## Abstract

**Purpose:**

To clarify the long-term effect of immunotherapy, the effect of adoptive activated T lymphocyte immunotherapy on advanced lung cancer was evaluated in terms of survival time. In addition, the performance status of cancer patients under immunotherapy was examined.

**Experimental design:**

Over 5 × 10^9^ alpha–beta T lymphocytes cultured ex vivo with an immobilized anti-CD3 antibody and interleukin-2 were injected intravenously into patients, once every 2 weeks for 3 months or longer. Follow-up of these patients was carried out using clinical records and by telephone interview questionnaire. Patients undergoing immunotherapy in immunotherapy clinics and those undergoing other anticancer therapies without immunotherapy in seven hospitals in Tokyo were enrolled in this study. Data were analyzed by a third-party statistician. Performance status was studied on another series of various cancer patients who underwent immunotherapy.

**Results:**

The overall median survival time of the patients with the best supportive care, which was obtained using Kaplan–Meier’s model, was 5.6 months, and those with immunotherapy alone, chemotherapy alone, and immuno-chemotherapy were 12.5, 15.7, and 20.8 months, respectively. Using Cox’ proportional hazard model, we examined the possible factors on survival time by univariate analysis. Then, the patients were stratified by gender and histological type for multivariate analysis. Significantly low hazard ratios were observed for immunotherapy and radiotherapy in males with squamous cancer; for chemotherapy and radiotherapy in male with adenocarcinoma; and for immunotherapy in females with adenocarcinoma. Addition of immunotherapy to chemotherapy resulted in a statistically significant decrease in hazard ratio in females with adenocarcinoma. Studies on the performance status (PS), determined according to the European Cooperative Oncology Group criteria, revealed a continuous high level of PS under immunotherapy until around 2 months before death, in contrast to the gradual increase of tumor marker level.

**Conclusions:**

The effectiveness of immunotherapy on advanced lung cancer is limited but may extend life span under certain conditions. Immunotherapy itself provided no clinical benefit by itself as compared with chemotherapy, but a significant additive effect of immunotherapy on chemotherapy was observed in females with adenocarcinoma. Moreover, immunotherapy can maintain good quality of life of the patients until near the time of death.

## Introduction

It is widely accepted that surgical resection of solitary tumors is the first-line treatment for primary lung cancer, followed by radiotherapy of localized cancers in some cases. For advanced lung cancer with extrapulmonary metastasis, various anticancer drugs have been administered with limited effects, and other effective treatments are expected to be developed.

In accordance with recent progress in immunology, cancer immunotherapy firstly emerged in the field of basic research, and lymphokine-activated killer (LAK), particularly those activated by interleukin-2 (IL-2), were actively studied using animal models by Rosenberg with his colleagues [1, 2] and other researchers [3–12] in the 1980s. Hopeful findings were then applied clinically with IL-2 injection for advanced cancers [13, 14], and immunotherapy was expected to be a new treatment strategy for cancer. However, the efficacy of immunotherapy was limited with frequent adverse reactions to IL-2, leading to hesitation for the LAK therapy. With the identification of cancer antigens in basic researches in recent years, several cancer-specific peptides, such as MUC-1, MART-1, TRP-2, gp100, NYESO, Her2/neu, and others, had been administered to cancer patients with an adjuvant, with or without dendritic cells. However, the clinical response rate has been evaluated to be low by a worldwide literature survey [15]. Tumor-infiltrating lymphocytes (TILs), which are obtained from tumor tissue and presumed to be sensitized in situ by cancer antigens, have been reported to have a good response rate. However, chances to isolate lymphocytes infiltrating in the surgically removed tumor tissues are limited in clinical practice. Peptides expressed on the cell surface of tumor-associated macrophages (TAMs) could be alternative tumor-specific antigens in immunotherapy [16–18]. Legumain, a member of the asparaginyl endopeptidase family, was considered to be overexpressed by TAMs, which may be a target for suppressing tumor growth. In vivo studies of mice supported this potential, but clinical approaches using TAMs are few at present. Tumor-specific proteins used in cancer vaccine therapy are expected to exert their cytotoxicity through specifically sensitized CD8^+^ T lymphocytes [19–21]. In contrast, gamma/delta T cells, which are a small portion of T cells, can attack cancer cells through the pathways different from those in alpha–beta T cells. NKG2D molecules on the surface of gamma/delta T cells bind to MIC A/B molecules of tumor cells, by a non-specific, non-MHC restricted mechanism, exhibiting cytotoxicity to target cells. Bisphophonate-stimulated and expanded gamma/delta T cells in adoptive immunotherapy are also the recent subject of clinical studies [22–25]. Randomized case–control studies of LAK therapy as an adjuvant therapy for postoperative lung cancer patients [26] and hepatic cancer patients [27] have been conducted; such patients showed a significantly higher postoperative survival rate than the control patients without the adjuvant therapy. On the basis of these studies, Seta Clinic Group started in 1999 the adoptive alpha/beta T cell immune therapy without IL-2 injection, for various types of cancer preferentially in the advanced stage with or without chemo- or radiotherapy, and good responses were observed in some cases without serious side effects [28–31]. According to the response evaluation criteria in solid tumor (RECIST), the effective response rate (CR/PR) of the immune therapy was determined to be low, but adverse reactions were hardly observed and patients generally showed a good performance status during the course of immune therapy. Because there are only few reports on a long-term follow-up of patients who underwent adoptive T lymphocyte immune therapy without IL-2 injection, we assessed survival time on the basis of evidence-based medicine, to be the end points of the effectiveness of immunotherapy alone and immunotherapy combined with chemotherapy or radiotherapy. The performance status of cancer patients receiving immunotherapy was also studied.

## Materials and methods

### Adoptive activated T lymphocyte immunotherapy

Peripheral blood mononuclear cells were harvested by centrifugation, and over 1 × 10^6^ harvested cells were cultured with an immobilized anti-CD3 antibody and IL-2 for 14 days, obtaining over 5 × 10^9^ lymphocytes on average. The cultured lymphocytes consisted of 61 ± 15% CD8^+^, 30 ± 15% CD4^+^ (CD4^+^:CD8^+^ ratio, 0.8 on average) and a small percentage of NK cells and NKT cells, indicating that CD8^+^ T lymphocytes proliferated more intensively than CD4^+^ T lymphocytes during the 2-week culture period [32]. These lymphocytes were substantially activated, showing increasing expression levels of IFN-gamma, TNF-alpha, LFA, and ICAM with cytotoxicity to Daudi’ and K562 cells with increasing E/T ratio (data not shown). Expanded CD8^+^—rich T lymphocytes were infused intravenously once every 2 weeks for at least 3 months. At the end of one course (six times) of immunotherapy, radiological findings of the tumor were evaluated on the basis of RECIST. When tumor was determined to be stable (SD) or showing a good response (PR, CR) on radiograms, the immunotherapy was continued. The median duration of immunotherapy was 6.1 months, and median frequency of lymphocyte infusion was in median 8.7 times. When disease progression was observed, immunotherapy was discontinued usually changing the approach to providing the best supportive care.

### Case enrollment

A committee, Lung Cancer Immunotherapy Evaluation Group (LITEG, Chief, S. Kudoh), was organized in 2007, consisting of the members of the Department of Chest Diseases in two university hospitals and six major hospitals in Tokyo who were interested in but had limited experience in immunotherapy, and members of Seta Clinic Group where immunotherapy has been conducted since 1999. One statistician without any clinical experience in the practice on cancer chemotherapy nor immunotherapy, participated in this study to analyze the obtained data.

All of the three hundred and fifty-five patients with primary non-small cell lung cancer patients at stage IIIb/IV with an ECOG performance status of 0/1, and who visited these institutions for the initial treatment of lung cancer from January 1, 2003 to December 31, 2006, were enrolled in this multicenter study as the control group. Patients with secondary (metastatic) lung cancer, double or multiprimary cancers cases or postoperative relapse were excluded. One hundred and ninety-two patients with primary lung cancer, the enrollment criteria of which are the same as those of control group, were treated by immunotherapy in Seta Clinic from October 1, 2002 to December 31, 2006 and were enrolled as the immunotherapy group. Among the 547 patients, 207 underwent chemotherapy alone (CT), 118 chemo-radio-therapy (CRT), 31 immunotherapy alone (IT), 132 immunotherapy with simultaneous chemotherapy (ICT), 27 immuno-chemo-radiation therapy (ICRT), and 25 best supportive care (BSC) were included in the analysis. As the number of patients who had underwent radiotherapy alone (*n* = 5) and immuno-radio-therapy (*n* = 2) were very small, they were excluded from analysis.

### Data on patient characteristics

Clinical data (e.g., age, gender, time of diagnosis, number and sites of metastases, starting time and duration of treatment, kinds of anticancer drugs, with or without radiotherapy [to primary lesion]), were obtained from clinical charts, recorded in a prepared form, and examined. Regarding end point data, it was carefully examined whether the patient was alive or deceased at the time of last visit to the hospital. When they were found to be alive at the time of their last visit or, in the case of no information available on their clinical course, a telephone inquiry was carried out to the patients’ homes to confirm the most recent living status of the patients. Observation duration and the final status of the patients were recorded and stored in a computer data base.

In the CT and ICT groups, the principal treatment regimen, consisted of administration of platinum-containing drugs with other various anticancer drugs given for 3 weeks or in a 1-month cycle of 4–6 courses. Gefitinib was allowed to be used in Japan by the health insurance system from September 2004 only as a second-line drug, and few patients were treated with it as the initial treatment drug in this study period. Bevacizmab was not used in the study period. In the immunotherapy groups (IT, ICT, and ICRT), most patients received immunotherapy as the final therapy following standard cancer therapy, and the interval between the start of chemotherapy and immunotherapy was 6.2 ± 7.2 months, on average. Some of the patients received immunotherapy alone for the reasons of high age, complication of interstitial lung fibrosis, and fear of severe side effects of chemotherapy. The characteristics of the patients in each treatment group are shown in Table 1.

**Table 1:** Patient characteristics in each treatment group

Patient characteristics	Total	BSC	IT	CT	ICT	CRT	ICRT
	*n* = 540	*n* = 25	*n* = 31	*n* = 207	*n* = 132	*n* = 118	*n* = 27
Age (years old)							
Median	65	77	71	65	60	69	60
Range (25, 75%)	(58, 73)	(70, 83)	(59, 78)	(59, 73)	(54, 67)	(61, 74)	(51, 67)
Gender							
M:F	366:174	17:08	21:10	134:73	79:53	97:21	18:09
(%)	67.8:32.2	68:32	67.8:32.2	64.7:35.2	59.8:40.2	82.2:17.8	66.7:33.3
Stage							
III:IV	166:374	8:17	8:23	47:160	21:111	65:53	17:10
(%)	30.7:69.3	32:68	25.8:74.2	22.7:77.3	15.9:84.1	55.1:44.9	63.0:37.0
Histology							
Aden:squa	374:126	13:11	20:6	148:43	111:12	63:47	19:7
(%)	74.8:25.2	54.2:45.8	76.9:23.1	77.5:22.5	90.2:9.8	57.3:42.7	73.1:26.9
PS							
0:1	322:218	15:10	16:15	131:76	63:69	78:40	19:8
(%)	59.6:40.4	60:40	51.6:48.4	63.3:36.7	47.7:52.3	66.1:33.9	70.4:29.6
Organ metastases							
Pleura (%)		0	47.4	6.3	23.3	0	13.6
Lung (%)		23.1	42.1	38.0	31.4	24.6	13.6
Brain (%)		38.5	21.1	27.5	29.1	42.1	45.5
Bone (%)		76.9	10.5	43.0	34.9	24.6	36.4
Liver (%)		23.1	5.3	14.1	9.3	8.8	0
Adrenal glands (%)		0	0	7.7	7.0	14.0	4.5
Others (%)		15.4	21.1	8.5	19.8	12.3	31.8
Mean no. of metastasis sites/patient		1.46	1.47	1.45	1.55	1.26	1.45
Treatment							
Platinum + %^a^	84.8	0	0	86.5	86.3	88.1	88.9
Gefinitib + %^b^	31.0	0	0	34.7	41.6	18.6	7.4
No. of IT infusions							
Median	8.7	–	8.0	–	6.0	–	7.0
Range (25, 75%)	(6.0, 10.0)	–	(6.0, 15.0)	–	(4.5, 10.0)	–	(6.0, 11.0)
Follow-up (mo.)							
Median	15.2	4.0	15.2	13.2	17.3	16.3	18.3
Range (25, 75%)	(8.1, 26.9)	(3.0, 6.0)	(7.6, 28.4)	(7.1, 26.4)	(11.2, 27.4)	(9.1, 29.4)	(13.2, 38.6)

*BSC* best supportive care, *IT* immunotherapy, *CT* chemotherapy, *ICT* immunochemotharapy, *CRT* chemoradiotherapy, *ICRT* immuno-chemo-radiation-therapy

^a^Platinum-containing regimens administrated as initial drugs

^b^Administrated as second-line drug

### Survival time calculation

The enrolled patients were followed up starting in early 2009. The median follow-up period of all the patients was 15.2 months with an interquarterly range between 8.1 and 26.9 months. According to information on their present status or date of death, survival time was calculated as the time from the start of treatment or from diagnosis in the case of BSC. Clinical data were collected from all institutions, summarized in a computer at the study center, and analyzed by a statistician who had no experience on cancer treatment nor immunotherapy.

### End point of the study

Overall median survival (OMS), 1-year survival rate (1-YS), and 2-year survival rate (2-YS) were obtained using Kaplan–Meier’s model as the primary end points of this study. The IT patients were examined on the basis of RECIST at the end of the 3rd month (after one course), and the results of which were compared with OMS.

### Statistic analysis

For the survival curves obtained using Kaplan–Meier’s model, the statistical difference between the treatment groups was determined by the Log-Rank and generalized Wilcoxon tests. Next, Cox’s proportional hazard model was applied to the analysis of the significance of the effectiveness of the treatment in each group. Initially, univariate analysis was carried out to examine the possible confounding factors, after which, multivariate analysis by stratification of the patients according to the confoundable basic factors, gender, and histological type, was conducted to study the significance of the hazard risk value of each treatment group and that of the combination effect of immuno-chemotherapy.

### Studies on performance status (PS) of the patients underwent immunotherapy

In additional series of 72 patients with various types of cancer, who received immunotherapy from January 1, 2008 to December 31, 2010, were examined on the time course of PS, assessed on the basis of the European Cooperative Oncology Group (ECOG) criteria, to determine the quality of life during immunotherapy. The types of cancer were as follows; 12 pancreas cancers, 11 lung cancers, 7 gastric cancers, 7 colon cancers, 6 esophageal cancers, 5 ovarian cancers, 4 breast cancers, 4 liver cancers, 3 prostate cancers, 2 uterine cancers, 2 pharyngeal cancers, and 9 other cancers. The PS score described on the clinical records on every immunotherapy date were reviewed and analyzed with tumor marker level.

## Results

From the background characteristics of the patients shown in Table 1, the number of males is approximately two times larger than that of females; the average age of BSC patients at the time of the first visit is older than those of other patients; and the number of adenocarcinoma (Ad) patients are nearly four times larger than that of epidermoid cancer (Ep) patients. At the start of each treatment, metastasis to other organs was noted on an average number of 1.47 per patient, including those with distant metastases to the bone, lung, brain, pleura, liver, adrenal glands, and other organs. Patients at stage IIIb included those with pleural metastasis or effusion associated with primary lung lesions during this study period.

**Fig. 1:**
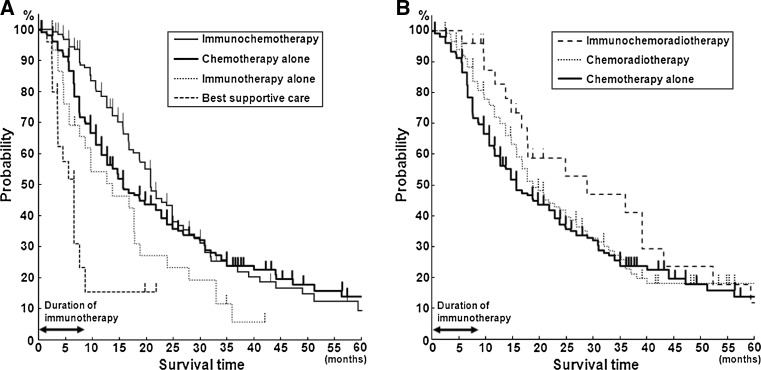
Comparison of survival curves among treatment groups using Kaplan–Meier model. **a** Comparison of BSC, IT, CT, and ICT groups, and **b** comparison of CT, CRT, and ICRT groups, showing an increasing probability of survival in this order

Figure 1a, b shows the overall survival curves and Table 2 shows the OMS, 1-YS, and 2-YS of the six treatment groups, in Kaplan–Meier’s model. The IT group showed a significantly better prognosis than the BSC group, and the OMS, 1-YS, and 2-YS of the IT group were 12.7 months, 54.1, and 15.5%, whereas those of the BSC group were 5.6 months, 15.5, and 0%, respectively, (Table 2). A statistically significant difference between the IT and BSC groups were found by both the Log-Rank (*p* = 0.03) and generalized Wilcoxon (*p* = 0.017) tests. The survival time of the IT group was calculated at the time from the start of immunotherapy, and when survival time was estimated from the time of diagnosis, the OMS of the IT group increased (16.7 months). Among the IT, CT, and ICT groups, the survival curve was better in order of the IT, CT, and ICT groups (Fig. 1a). Among the CT, CRT, and ICRT groups, the ICRT group showed the most favorable survival curve (Fig. 1b). The difference in survival time between the CT and ICT groups was found to be statistically significant by the generalized Wilcoxon’s test (*p* = 0.004), but not by the Log-Rank test (*p* = 0.18). No significant difference was observed between the CRT and ICRT groups.

**Table 2:**
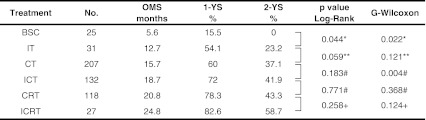
Overall median survival, 1-year survival rate, and 2-year survival rate, and *p* value

*IT versus BSC, **IT versus CT, ^#^ICT versus CT, ^##^ICT versus CRT, ^+^ICRT versus CRT

Note in Fig. 1a, that the survival probability 12 months after the start of treatment showed a slight but significant difference between the CT and ICT groups (*p* < 0.05), whereas no significant difference was observed at 24 months. The median frequency and duration of immunotherapy in this study were 8.7 (range in quartile, 6.0, 10.0) infusions and 6.1 (range in quartile, 3.0, 15.2) months, respectively, indicating that immunotherapy was no longer administered after 9 months on average and the effectiveness disappeared after 2 years.

Cox’s proportional hazard model was applied to determine the significance of suspected confounding factors. Hazard ratio, 95% CI, and the *p* value of hazard ratio for gender, histological types, age, stage of lung cancer, and PS of all patients are shown in Table 3. Gender, histological type, and the stage of lung cancer were found to be significant variates, among which the stage of cancer was considered to be a second-level variant that may change during the course of the disease.

**Table 3:** Univariate analysis by Cox’ proportional hazard method

Variate	Hazard ratio	95% CI, (upper and lower)		*p* value
Gender				
Male	1			
Female	0.68	0.59	0.78	<0.0001
Age				
≥70	1			
<70	0.87	0.77	0.99	0.384
Histology				
Squamous	1			
Adeno carcinoma	0.89	0.77	1.02	0.1015
Stage				
IV	1			
III	0.83	0.72	0.95	0.006
PS				
0				
1	1.05	0.93	1.18	0.3976
Chemotherapy				
No	1			
Yes	0.69	0.57	0.85	0.0009
Radiotherapy				
No	1			
Yes	0.81	0.69	0.93	0.003
Immunotherapy				
No	1			
Yes	0.81	0.93	1.18	0.0016

For the multivariate analysis of Cox’ proportional hazard model, the patients were stratified according to gender and histological type. The hazard ratio with 95% CI and *p* value of each therapy alone were estimated and are shown in Table 4. In the male/squamous cancer group, a significant *p* value was found in the IT group. In the male/adenocarcinoma group, CT was a significant variant, and in the female/adenocarcinoma group, IT was a significant variant.

**Table 4:** Multivariate analysis: Cox’s hazard ratio and *p* value for treatment in three stratified groups

Group	Treatment	Hazard ratio	95% CI	*p* value
Male/squamous	IT	0.75	0.51, 1.04	0.088
	CT	0.75	0.52, 1.13	0.16
	RT	0.71	0.55, 0.92	0.009
Male/adenocarcinoma	IT	0.386	0.32, 1.03	0.102
	CT	0.63	0.48, 0.85	0.003
	RT	0.79	0.63, 0.98	0.032
Female/adeno.	IT	0.73	0.55, 0.94	0.016
	CT	0.76	0.48, 1.40	0.339

Next, the additive effect of IT or RT on CT was tested (Table 5). IT added to CT showed a statistically significant additive effect in the female/adenocarcinoma group (*p* = 0.038). RT added to CT showed statistically significant *p* values in male/squamous (*p* = 0.004) and male/adenocarcinoma (*p* = 0.002).

**Table 5:** Additive effect of immunotherapy and radiotherapy to chemotherapy

Subgroup	Hazard ratio	IT on CT 95% CI	*p* value	Hazard ratio	RT on CT 95% CI	*p* value
Male/squamous	0.915	0.618, 1.276	0.621	0.67	0.508, 0.878	0.004
Male/adenocarcinoma	0.869	0.721, 1.041	0.128	0.736	0.596, 0.899	0.002
Female/squamous	0.818	0.374, 1.604	0.567	nd	nd	nd
Female/adenocarcinoma	0.758	0.572, 0.985	0.038	nd	nd	nd

*nd* not determined due to a small number of patients in the subgroup

**Fig. 2:**
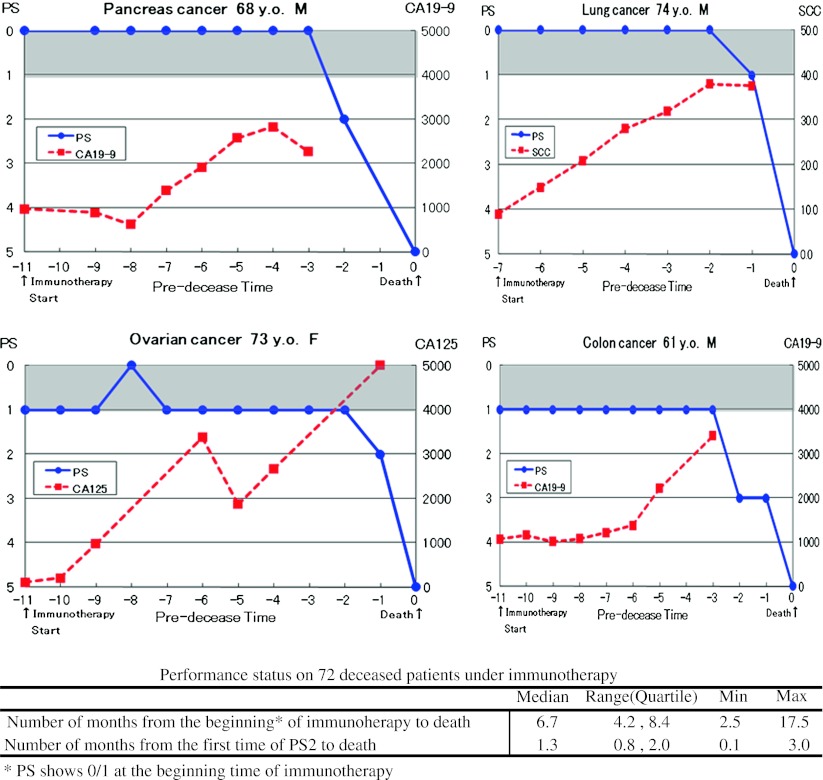
Performance status (PS) and tumor marker levels from staring time of immunotherapy to death. Illustrations of the disease course on 4 representative patients, and a summarizing table of PS on 72 cancer patients

Regarding PS during the course of immunotherapy of the patients who died finally, another series of patients with primary cancer of various organs was examined. Patients with lung cancer (11), pancreas cancer (12), gastric cancer (7), intestine cancer (7), esophagus cancer (6), ovarian cancer (5), breast cancer (4), liver cancer (4), prostate cancer (3), and other cancers (13) were included in this series. The patients who showed PS 0/1 at the start of immunotherapy, maintained this good status for a median of 5.4 months (4.2 and 8.4 months in quartiles), but after transition to PS 2, the patients rapidly deteriorate and died after 1.3 months (median) and 0.1 and 3.0 months of the minimum and maximum, respectively (Fig. 2).

## Discussion

This study is a historical cohort study of the autologous activated T lymphocytes therapy, using alpha–beta T cells, without IL-2 injection. Retrospective studies generally include various confounding factors that may mask the true outcomes. In this study, we examined the data of a large number of patients treated in the past to determine overall survival, and analyzed them, avoiding possible confounding factors by stratifying the patients for significance tests.

As the initial step, the patients’ survival time after diagnosis of the disease or initiation of immunotherapy was evaluated using Kaplan–Meier’s model to obtain overall survival curves and to calculate OMS, 1-YS, and 2-YS. A statistically significant difference in survival time was found between the BSC group and the IT group, indicating that IT alone can increase the life span of lung cancer patients. The natural history of inoperable lung cancer patients was reported by Hyde [33] in 1970, in which the median survival time was noted to be 22 weeks. In the later studies on the effect of cancer chemotherapy, the median survival time of the no-drug control group was estimated to be in the range from 4.2 to 5.9 months [34–39], which is nearly the same as that obtained in the present study (5.6 months). The natural course of lung cancer in recent years may be longer, as the disease is diagnosed in an earlier stage with better performance status than in the past, even though survival time is still shorter than 6 months, similar to that in the present study. OMS of 12.7 months in the IT group noted in this study may indicate the extension of patients’ life span by immunotherapy alone. However, the efficacy disappeared after 2 years, in patients who received immunotherapy limited to, 8.7 infusions and for 6.1 months in medians, as in this study (Fig. 1). A longer treatment may be required to extend further the patients’ survival.

The estimated overall survival time, however, may differ according to the characteristics of the patients enrolled in the studies. Univariate analysis using Cox’s proportional hazard model indicated that hazard ratio and its *p* value may be significantly affected by the different ratios of male to female, epidermoid to adenocarcinoma, and stage IIIb to stage IV. In a previous study of liver cancer, females were found to have a better prognosis than males [40], and a large-scale survey of postoperative survival time for lung cancer in Japan also yielded a higher 5-year disease-free survival rate in females than males [41]. In the present study as well, a significantly longer survival time was observed in females than in males in each treatment group. Gender difference might be considered as a significant factor in every follow-up study of lung cancer patients. Stratification of the data by gender and histological type using Cox’ proportional hazard model revealed significant *p* values for males with squamous carcinoma in the IT group, males with adenocarcinoma in the CT group, and females with adenocarcinoma in the IT group. The combined effects of CT and IT were not observed in the squamous cancer group but were observed in the adenocarcinoma group, showing a statistically significant additive effect of IT on females with adenocarcinoma. As expected, the survival time well reflected the results of RECIST in this study (data not shown), and the responses of tumor at the end of the 3rd month of treatment were considered to be a good predictor of the subsequent survival time. It was also shown, however, that immunotherapy resulted in a rather low response rate when evaluated with RECIST, but kept the disease stable, which may improve survival time with a maintained high performance status. In this regard, quality adjusted life year (QALY) could be a better landmark for evaluating the efficacy of IT in future studies.

Although adoptive activated T lymphocyte immunotherapy is a non-specific therapy without sensitization by cancer-specific peptides, immunotherapy indeed extended the patients’ life significantly under certain conditions. Regarding the question on why this non-specific adoptive immunotherapy is effective, it may be considered firstly as a possible mechanism that sensitization against tumor cell antigens is naturally induced in a patient’s body although it may be in a small percentage of T cells, but these sensitized T lymphocytes in the patients’ peripheral blood can be increased in number and activated during the 2 week ex vivo culture process. Actually, in our present study, CD8^+^ T lymphocytes actively proliferate and become the predominant cell type (over 60% on average) during the culture period, showing promoted killer activities and a decrease in the number of regulatory T lymphocytes, which suppress tumor immunity [32]. These infused activated CD8^+^ T cells may contribute to the enhancement of the suppressive and cytotoxic activities of a patient’ s immune system, during the repeated infusions of immune cells. Secondly, the infused adoptive T lymphocytes including central memory CD8^+^ T cell may retain, and with time, be accumulated in the body resulting in an increase in the total number of CD8^+^ T cells with an inversion of CD4:8 ratio in the peripheral blood, which was also observed in our other clinical studies (data not shown). Thirdly, the absence of adverse reactions to immunotherapy without IL-2 injection may make long-term treatment possible which is necessary to achieve significant efficacy of immunotherapy.

Further clinical studies will be required to confirm the present findings by a large-scale prospective study and to examine in detail the laboratory test results and the tumor-suppressive immunological status of the patients under repeated adoptive activated T lymphocyte treatments.
